# Breed- and Line-Dependent Severity of Inflammation and Necrosis Syndrome in AI Boars, and the Related Risk of Inflammation and Necrosis in Their Progeny

**DOI:** 10.3390/vetsci12100967

**Published:** 2025-10-09

**Authors:** Sabrina Becker, Eva Kochendoerfer, Josef Kuehling, Katharina Gerhards, Mirjam Lechner, Silvia Zinner, Matthias Lautner, Gerald Reiner

**Affiliations:** 1 Department of Veterinary Clinical Sciences, Clinic for Swine, Justus-Liebig-University, 35392 Giessen, Germany; 2 UEG Hohenlohe-Franken, Kraussenklinge 1, 97996 Niederstetten, Germany; 3 Besamungsverein Neustadt a.d. Aisch e.V., Franz-Ehrsam-Weg 1, 91413 Neustadt an der Aisch, Germany

**Keywords:** swine, animal welfare, inflammation, selection

## Abstract

**Simple Summary:**

Swine Inflammation and Necrosis Syndrome (SINS) can be detected in different body regions of pigs, such as the tail, ears, teats, or claws, and is an important indicator for health and welfare. Besides environmental influences, genetic factors play a major role in the development of SINS. In this study, we examined boars from different breeds kept at an artificial insemination (AI) station and compared their visible signs of SINS with those of their offspring. We found clear differences between breeds and lines, and a strong association between the severity of SINS in boars and in their piglets. Offspring of boars with the highest SINS scores also showed the most pronounced signs of SINS. However, some changes in heels and claws in the boars were mainly caused by environmental factors. Our findings suggest that pre-selecting AI boars with fewer signs of SINS could help reduce the occurrence of SINS in piglets and thereby improve their health and welfare.

**Abstract:**

Animal-based measures, such as detecting inflammation in areas like the tail, ears, teats, coronary band, heels and claws (Swine Inflammation and Necrosis Syndrome, SINS), are used to monitor animal health and welfare. When parameters deviate from the established range, these measures enable prompt action to adjust husbandry practices, feeding regimens and management strategies. In addition to environmental factors, genetics have been shown to play a key role in inflammation and necrosis processes, and selection can reduce the severity of the disease. This study examined whether different breeds of AI boar exhibit different signs of SINS and how these signs are associated with SINS in their offspring when they are suckling piglets and weaners. Initially, 286 AI boars of 7 breeds from a German artificial insemination center were evaluated for SINS. The following parameters were assessed: tail base, tail tip, ears, skin, scrotum, coronary bands, heels and claws. Subsequently, 23 Pietrain and Duroc boars were used in combination with a Topigs DL sow line. The progeny of the AI boars was evaluated as suckling and weaned piglets, with the assessment framework encompassing SINS traits. The results revealed significant differences between the breeds and lines, as well as a strong correlation between the SINS phenotypes of the AI boars and the SINS scores of their offspring. The offspring of the 25% most extreme boars exhibited a 17% variation in SINS scores. This association was particularly evident when comparing the boars’ tail base. However, the development of the boars’ heels and claws was found to be significantly influenced by mechanical environmental factors and not associated with the piglets’ scores. These findings imply that heritable, endogenous processes, as proposed for SINS, also visibly impact the phenotype of the AI boar. This study’s fundamental premise suggests that pre-selecting AI boars could mitigate the occurrence of SINS and enhance piglet health and welfare.

## 1. Introduction

The ethical and social obligation to prioritize the health and welfare of animals is well-established [1,2]. The behavior, welfare and health of animals are determined by a combination of hereditary traits and the conditions in which they are kept on a farm. In order to assess them, it is necessary to employ a combination of resource-based measures (RBMs [3]) and direct clinical observation of the animals themselves (animal-based measures, ABMs [4,5,6]).

The Swine Inflammation and Necrosis Syndrome (SINS) offers a valuable opportunity to record Animal Based Measures (ABMs). SINS is a multifactorial condition characterized by inflammatory and necrotic lesions on various peripheral body parts of pigs, including the tail, ears, teats, coronary bands, heels and claws. The aforementioned lesions have been observed in a variety of age groups, including newborns [7], suckling piglets, weaners, and even finishers [8], with the majority occurring without overt signs of trauma, suggesting an endogenous pathogenesis [9]. Research conducted in Germany [8,10,11], France [12] and the Netherlands [6] has demonstrated SINS prevalence to be 30–40% on average, with the most severe symptoms manifesting in weaners, followed by suckling piglets. The presence of SINS signs in finishers was also detected, and these were found to be associated with the condition of the sow and the husbandry practices from weaning to finishing [8]. The hypothesis that an endogenous primary event is responsible for the observed phenomenon is based on two factors. Firstly, the simultaneous occurrence of inflammation in different body parts. Secondly, the detection of SINS signs in new-born piglets within two hours of birth [7]. A substantial proportion of the newborn piglets exhibited alterations to the tail base, claw wall and heels, with over 80% of the subjects demonstrating such changes. In 65–87% of animals, the coronary bands, teats, face and ears were affected. The analysis revealed an average of six out of nine body parts to be affected, with no instances of piglets being found to be free of pathological signs. Histopathological analysis of the piglets revealed an intact epidermis, but also numerous granulocytes, macrophages and lymphocytes in the subepithelial connective tissue. Such an accumulation is not expected until 4–7 days after an inflammatory trigger [13]. In suckling, weanling and finishing piglets with SINS, signs of inflammation in areas with clinical alterations were also evident, including vasculitis, intima proliferation and thrombotic changes [8]. Systemic inflammation and hemostasis disorders were evident in clinical chemical analyses of serum from piglets [14]. A positive correlation was identified between higher degrees of clinical SINS and increased numbers of monocytes and neutrophils. The process of blood coagulation was found to be altered in weaners, and thrombocytopenia was observed in fatteners. Furthermore, acute-phase proteins, most notably C-reactive protein and fibrinogen, were found to be elevated in the serum. Serum liver enzymes demonstrated only minor alterations. In general, aspartate transaminase levels were found to exceed physiological limits and increased in parallel with SINS scores in fattening pigs. The generalized inflammatory nature of SINS was further demonstrated by comparing the liver gene expression of piglets with and without obvious signs of SINS [15,16]. A number of pro-inflammatory, acute-phase and stress-response genes were identified in piglets with SINS. The transcriptomic findings were corroborated by the metabolome analysis results [15]. The observed deviations in metabolic metabolites, notably those pertaining to lipid metabolism in piglets exhibiting pronounced symptoms of SINS, substantiated the transcriptomic abnormalities and signalled hepatic stress induced by metabolism. The results of this study provided the first evidence of an inflammatory process induced in the livers of piglets with SINS, accompanied by deranged lipid metabolism. Gerhards et al. [16] further characterized the inflammatory aspect of SINS, emphasising the importance of inflammatory and defence genes, such as CRP, as well as other candidate genes, including S100A12, GYPA, and LIPK. The presented studies demonstrate that SINS is not merely a local skin condition, but also involves hepatic inflammation and metabolic dysregulation. A comprehensive hypothesis on the pathogenesis can be found in Reiner et al. [9]. The syndrome has been increasingly recognized in recent years, raising concerns in terms of animal welfare, productivity and genetic susceptibility [6,9,10,11,12].

In order to combat the symptoms, husbandry, feeding and management conditions must be taken into account [8], in particular, opportunities for thermoregulation and an optimal water and feed supply [9]. However, recent studies have identified a genetic predisposition for this disease. In the context of standardized environmental conditions, substantial sow effects were described from an evaluation of a population of 4725 piglets from four distinct sow lines [17]. The impact of sow lines on coronary bands, tail and ear varied between 3 and 14% of phenotypic variance. Additionally, observations from breeding farms indicate the presence of disparities amongst individual boars with regard to the frequency and severity of SINS lesions in their offspring, even under conditions of identical housing, feeding, and management conditions [18]. Litters of Duroc boars exhibited a significantly lower incidence of SINS signs, with a 59% reduction observed compared to Pietrain boars. Furthermore, the progeny of the most favourable Pietrain boars demonstrated a 37% decrease in SINS signs, while the least favourable Pietrain boars exhibited the highest incidence of these signs. The presence of necrosis was not detected in the Duroc progeny. However, necrosis was detected in 4.4% and 20.1% of the offspring from the most and least favourable Pietrain boars, respectively. Leite et al. [11] observed a 75% prevalence of affected piglets among the offspring of Pietrain boars, a 67% prevalence among those of Large White boars, and a 42.5% prevalence among those of Duroc boars. These findings confirm that Pietrain litters are more susceptible to SINS than Duroc litters. The direct heritabilities of 0.24 for the tail, 0.33 for the teats, 0.34 for the claws and 0.26 for SINS in 3-day-old suckling piglets indicate that a reduction in SINS incidence through genetic selection is indeed feasible. Gene loci associated with SINS appear to be widely distributed across the pig genome [19]. Furthermore, functional gene variants [20] and corresponding candidate genes from transcriptomic and genomic studies [16] were identified. These results suggest that SINS and its associated traits are polygenic traits, which makes direct selection based on gene markers difficult.

However, as proposed by Leite et al. [11], the implementation of selection strategies against SINS would result in a substantial reduction in inflammation and necrosis, accompanied by a decline in biting behavior during later stages of life. Concurrently, this approach would lead to augmented birth and weaning weights, an increase in the number of piglets, and, in turn, an enhancement in overall animal welfare [11].

Although the heritability of SINS [11] and the influence of boar effects on offspring [18] have been established, the variability in phenotypic expression among boars remains unclear. It is therefore unresolved whether the boar phenotype allows conclusions regarding the burden in their progeny. Demonstrating such associations could strengthen hypotheses on the pathophysiology of SINS, supporting the assumption that boars with specific characteristics also transmit a genetic predisposition. Because phenotypic traits can be recorded rapidly and non-invasively, their documentation could represent a practical complement to genetic selection protocols and contribute to breeding strategies aimed at reducing the occurrence of SINS. Moreover, integrating boar phenotype assessment into routine AI examinations may provide a predictive criterion for offspring robustness.

The present study aimed to determine whether boars used for semen collection in artificial insemination programs (AI boars) of different breeds and lines differ in clinically assessable SINS signs, and whether distinct anatomical regions of the boar are differentially correlated with SINS manifestations in suckling piglets and weaners. The methodology is non-invasive, simple, and easy to implement, and its application could enhance the understanding of SINS indicators in AI boars while supporting selection against SINS at the phenotypic level.

## 2. Materials and Methods

### 2.1. Experimental Design and the Tested Animals

The experiment was conducted in two parts. Initially, a total of 286 AI boars from a German insemination station were examined for the presence of inflammation and necrosis. The boars were derived from 16 breeding lines within seven breeds, including Pietrain (PI), commercial Large White (LW), herdbook Large White (LWH), Duroc (DU), Landrace (LR), PIxDU, and Angler Saddle back (AS). The breeding lines were used anonymously, as the number of animals available did not allow for any general conclusions to be drawn about them. The boars were accommodated in three stables, each with a distinct flooring type. The examinations were carried out without direct physical contact, as part of the boars’ normal daily routine. Photographic documentation was undertaken of the subjects’ tails, ears, skin, scrotum, coronary band, heels and claws, both in their pens and during the process of semen collection. The evaluation was conducted at a subsequent point in time. No intervention was performed on the boars.

In the second part of the trial, semen from a selection of 23 Pietrain and Duroc AI boars was used to inseminate 31 sows at the Oberer Hardthof teaching and research station as part of the farm’s normal piglet production process. Each sow was inseminated with mixed semen from two different boars. The mating pairs did not deviate from the farm’s standard procedures. The distinguishing factor pertained to the utilization of mixed semen from two boars per sow by the farm, a strategy employed to enhance the sow-boar combinations within a manageable number of piglets, thereby reducing the overall variability. This finding indicates that the presence of piglets from two distinct boars was observed in each litter. It is evident that not all potential boar combinations could be produced in the experiment; however, the number of sow-boar combinations was doubled. The assignment of piglets to specific boars was conducted through the utilization of paternity tests. This resulted in 43 litters (farrowed from May to September 2023), of which 435 average (the 10 most average piglets per litter in terms of physical development and physical integrity were selected) and anatomically intact piglets were assessed on day 3 of life and at weaning (day 28). The piglets were photographed as part of the farm’s routine zootechnical measures (iron supplementation, tail docking on day 3, weaning on day 28) in such a way that the tail, ears, teats, coronary bands, heels and claws could be evaluated later. Within this framework, the piglets underwent tail docking to prevent subsequent injuries from tail biting. In order to facilitate the paternity testing of the piglets, DNA was extracted from the docked tail tips of the piglets and the sperm samples from the boars. No experimental manipulations were performed on the boars and piglets. The experiment consisted of non-invasive examinations only. The only procedure was the visual collection of clinical scoring results. Consequently, the experiment was not categorized as a case of animal testing by the responsible animal welfare officers (file reference: JLU_kTV_8_2025).

### 2.2. The Maintenance and Feeding of the AI Boars

The AI boars examined were sourced from an artificial insemination station, the purpose of which is the production of fresh semen for the purpose of artificial insemination. At the time of the study, the station housed approximately 300 pre- and post-pubertal boars of various breeds and lines, including representatives of the Piétrain, Duroc, herdbook Large White and commercial breeds, Large White, Landrace, Angler Saddleback and PIxDU.

The boars were accommodated in three hermetically sealed and hygienically separate barn buildings, with each boar being housed in its own pen. Access to each pen was restricted to a hygiene sluice. The boar pens, with a mean floor area ranging from 6 to 7.6 m^2^, were arranged in double rows. The pen walls were composed of vertical steel bars, which enabled the boars to both see and smell their conspecifics.

The swine were provided with food manually by employees via troughs on a daily basis following the semen collection period. The animals were fed a complete feed in pellet form consisting of barley, soybean meal, wheat, wheat bran, green meal and soybean meal. The following substances were added to the mixture: minerals, synthetic amino acids (lysine and DL-methionine), and vitamins (A, D3, E, C and B1). This analysis yielded the following composition: 19% crude protein, 2.25% crude fat, 6.5% crude fiber, 8.1% crude ash, 1.4% lysine, 0.5% methionine, 1.2% calcium, 0.9% phosphorus and 0.4% sodium, with a metabolisable energy of 11.4 MJ/kg. The boars were provided with 2 to 3 kg of the product on a daily basis, the quantity being adjusted according to their respective body weights, in accordance with the recommendations of the German Agricultural Society (DLG). The product contains 450 g of crude protein, 380 g of digestible protein, 40 g of lysine and 30 MJ of energy. Water of drinking quality was provided ad libitum via nipple drinkers.

The individually pre-vaccinated boars were vaccinated once in quarantine against parvovirus and erysipelas (Parvoruvac, Ceva, Düsseldorf, Germany), as well as against PCV 2 and Mesomycoplasma hyopneumoniae (CircoMax Myco, Zoetis, Munich, Germany). Subsequent to this, the boars were administered a booster vaccination against parvovirus and erysipelas on a six-month basis using the aforementioned vaccine. The health status of the AI boars was subject to weekly monitoring, encompassing direct and indirect pathogen detection for a range of diseases, including Classical swine fever, African swine fever, Brucellosis, Aujeszky’s disease and Porcine reproductive and respiratory syndrome virus (PRRSV). The subjects were subjected to regular testing for Leptospirosis, with tests conducted on a quarterly basis. The pig health service monitored them for PRRSV (no antigens or antibodies). The boars were subjected to regular washing procedures, and their claws were meticulously maintained. Semen was collected from each boar on average two to three times per week.

Barn A was equipped with 79 fixed pens, with a surface area of 7.6 m^2^. Straw was utilized as bedding and was replaced every other day. In the experiment, barn B, which contained 150 pens and measured 6 m^2^, was partially perforated with straw bedding. The barn designated C, which contained 120 boxes with a surface area of 6 m^2^ each, was equipped with a layer of deep litter, measuring approximately 40 cm in thickness, composed of sawdust.

### 2.3. The Maintenance and Feeding of Sows

The sows were of a Topigs x German Landrace crossbred strain. The insemination of all sows was conducted using artificial means. At the breeding center, the sows were housed in compartments with concrete slats until day 28 of their pregnancy. The animals were provided with liquid feed in a long trough via an automatic feeder (Spotmix, Schauer, Prambachkirchen, Austria). Water was available for consumption via Aqua Level drinkers.

Within the designated waiting area, the sows were accommodated in a 145-square-meter compartment constructed from concrete slats, with separate areas designated for lying down. The animals were provided with unlimited quantities of feed. Water was made available to the subjects via nipple and Aqua Level drinkers.

The sows were vaccinated against erysipelas and parvovirus on day 14 of lactation (Porcilis Ery+Parvo, MSD Animal Health, Munich, Germany). Two weeks prior to farrowing, the animals were vaccinated against *Clostridium perfringens* (Clostriporc A, IDT, Dessau, Germany). The SPF herd was non-suspected of the following swine pathogens: Porcine reproductive and respiratory syndrome (PRRS), *Actinobacillus pleuropneumoniae*, *Lawsonia intracellularis*, *Brachyspira hyodysenteriae* and *Brachyspira pilosicoli*. During the study period, no cases of exudative epidermitis were observed.

Within the designated farrowing pen, sows and their suckling piglets were housed in enclosures measuring 4.8 m^2^, characterized by plastic slatted flooring. The sows were confined within a farrowing crate that was equipped with a flat lying surface. The sow’s floor was composed of cast-iron slats. The provision of feed was facilitated by a Spotmix feeder (Schauer, Prambachkirchen, Austria) that was situated within the trough. The animals were provided with water via nipple drinkers and mother-and-child drinking troughs.

The composition of the feed for pregnant sows was as follows: 12.5% crude protein, 2.8% crude fat, 7.0% crude fiber, 4.4% crude ash, 0.66% calcium, 0.46% phosphorus, 0.15% sodium, 0.7% lysine, 0.18% methionine and an energy content of 12.02% ME MJ/kg. The lactation diet was composed of the following ingredients: 16.0% crude protein, 3.3% crude fat, 5.0% crude fiber, 6.71% crude ash, 0.79% calcium, 0.54% phosphorus, 0.21% sodium, 0.94% lysine, 0.3% methionine, and an energy content of 12.71 MJ ME/kg.

### 2.4. Paternity Testing

The paternity tests were based on genetic matches between the offspring and the boars. The piglets underwent tail docking the day after the clinical evaluation. The methodology outlined by Kuehling et al. [18] was adhered to in the extraction of DNA from the docked tail tissue. The genotyping process involved the utilization of 14 microsatellites (SW936, S0155, S0226, S0227, S0026, S0225, SW240, SW951, SW911, S0101, SW72, SW24, SW632, and Swr1941), which were distributed across two multiplex polymerase chain reaction (PCR) reactions. Microsatellite allele determination was achieved using capillary gel electrophoresis, as previously described by Kuehling et al. [18].

### 2.5. Clinical Evaluation of AI Boars and Piglets

The clinical signs were documented in boars and piglets by means of a digital camera (Canon EOS DC 8.1 V, Canon, Willich, Germany) in accordance with a standardized scheme for the subsequent detailed evaluation of the images (Windows Media Player, Version 12, Microsoft GmbH, Germany).

**Figure 1:**
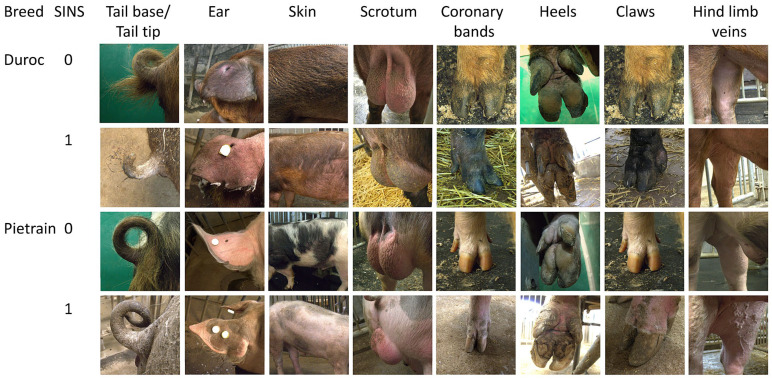
Examples of the evaluation of SINS-associated traits in AI boars, categorized according to the presence (1) or absence (0) of SINS signs in Duroc and Pietrain boars. Photos: Eva Kochendörfer.

The assessment of inflammation and necrosis in AI boars was conducted in accordance with the SINS grading scheme, as outlined by Reiner et al. [9]. Examples of unloaded body parts (score 0) and changes classified as SINS signs (score 1) are shown in Figure 1, separated into Duroc and Pietrain boars. For the tail base and the remainder of the tail, inclusive of the tail tip, binary records were made of the presence or absence of bristles (i.e., the absence of bristles was recorded as “no bristles”), as well as the presence of swelling, redness, exudation, necrosis or bleeding. In the event of its presence, the trait was assigned a rating of 1, whereas a rating of 0 was assigned in the absence of the trait. In addition, a thorough examination was conducted to ascertain the presence of ring-shaped indentations on the tail.

The ears were examined separately at the base, margin and tip for the absence of bristles, redness, swelling, exudation, necrosis and bleeding. Vein congestion was also a factor that was given full consideration. The absence of bristles and venous congestion were classified as minor or severe. A meticulous examination of the integument of the body surface, in addition to the scrotum and coronary bands, was conducted for the absence of bristles, swelling, redness, rhagades, exudation, necrosis and bleeding.

Photographs were taken of the heels of the animals at the time of the semen collection to score for hemorrhages, cracks and detachments. A thorough evaluation was also conducted to determine the presence of any cracks or hemorrhages in the claws. Furthermore, the veins of the hind limbs were examined for signs of congestion.

The clinical assessment of inflammation and necrosis in suckling and weaned piglets was conducted in a slightly modified manner, as outlined by Reiner et al. [17]. The assessment of the piglets was conducted on the third day of life and on the day of weaning, with the objective of ensuring comparability with other studies and because the clinical symptoms were clearly visible in all previous studies during this period [21]. Initial assessments were conducted to evaluate clinical changes at the base and tip of the tail, ears, teats, coronary bands, heels and claw wall. However, the assessment undertaken in this study was more detailed than that carried out by Kühling et al. [7,18]. The following clinical characteristics were considered and scored as follows: characteristics that were not visible received a score of 0, while characteristics that were visible received a score of 1. The tail was subjected to a thorough examination to ascertain the presence of bristles, swelling, redness, exudation, necrosis, bleeding and ring constrictions. The tail base was examined separately for the presence of bristles, swelling, redness, exudation and clinical signs of necrosis. The ears were examined for the presence of bristles, redness, exudation, necrosis and congested veins. The anatomic regions of the auricle, the anterior aspect of the ear, and the posterior aspect of the ear were given particular focus. The categorization of these regions was conducted into two distinct groups, namely mild and severe findings, respectively. The teats were examined for indications of swelling, redness, crusting and necrosis. The coronary bands were examined for indications of swelling, redness, exudation and necrosis. A comprehensive evaluation of the heels was conducted to determine the presence of swelling and bleeding. Subsequent to this examination, the heels were graded according to the severity of the conditions present and the extent of detachment. A thorough investigation was undertaken on each claw to determine the presence of any indications of bleeding, horn detachment, cracks or clefts.

All assessments were carried out by an experienced person on the basis of the photographs. Photographic documentation enables precise and reproducible assessment of SINS signs while minimizing handling time and animal stress. Images can be re-evaluated, archived, and used for training or digital analysis, thereby enhancing standardization and objectivity compared to live scoring. The assessments were conducted prior to the availability of the paternity test results, which precluded the possibility of assigning the piglets to the boars at the time of the assessment.

### 2.6. Statistics

The statistical analysis was conducted using IBM SPSS Version 27 (IBM, Munich, Germany). The prevalence of SINS traits in AI boars was calculated using a generalized linear mixed model (binomial, logit function) based on the binary distribution of the traits. The breed of the boar and the floor type were designated as fixed effects, the barn as a random effect, and the age of the boar as a covariate. In order to facilitate a clear comparison between AI boars of different breeds, the prevalence was also calculated as percentage of the mean value. The mean value was then utilized to establish a hierarchical ranking of the boar breeds.

The findings were then weighted and added up according to the corresponding organ, thus producing an organ score. Consequently, the boars were assigned scores for tail base, tail tip, ears, skin, scrotum, coronary bands, heels and claw wall. The following body parts were the subject of assessment in the study of suckling and weaned piglets: the tail base, tail tip, ears, teats, coronary bands, heels and claws. The body part scores were then aggregated to derive the SINS score. The hypothesis was that the mechanical environmental effects on the feet would be stronger than on the heels and claws. For the purpose of this study, an additional SINS score for the feet was calculated, with the heels and claws excluded from the analysis.

The metric body and SINS scores, which were Z-transformed (mean 0, standard deviation 1) to enhance the comparability of the data, were also analyzed using the aforementioned generalized linear mixed model (normal distribution, link function: identity) to calculate the effects of boar breed. The mean values were represented as bars, whilst the 95% confidence interval was indicated by whiskers.

Spearman’s rank correlation coefficient was utilized in order to calculate the correlation coefficients. The significance levels of the findings were represented as follows: It is indicated by the following abbreviations that the probability is less than 0.05, 0.01 or 0.001, respectively: *p* ≤ 0.05, *p* ≤ 0.01 or *p* ≤ 0.001.

The study of the boar’s offspring was only possible using Duroc and Pietrain boars. The selection of these items was conducted at random. The initial presentation of the prevalence of SINS traits in suckling and weaned piglets, along with their body scores, was of a purely descriptive nature.

The influence of the boar breed on the SINS traits of the piglets was calculated using a generalized linear mixed model. The boar line within the breed was designated as a fixed effect, while the sow and the boar herd were assigned as random effects. The age of the boar, the number of piglets born per litter, and the birth weight of the piglets were considered as covariates. The results were presented as mean values with standard errors. Statistically significant results were defined as *p*-values ≤ 0.05. The effects of the boar line were presented using data from the same model. The mean values were presented together with the 95% confidence interval.

The 23 AI boars with offspring data were additionally divided into three groups, with group 1 containing the 25% of boars with the lowest SINS-feet scores, group 3 containing the 25% of boars with the highest SINS-feet scores and group 2 containing the remaining 50% of boars. A similar grouping to that based on the SINS traits was carried out for all body parts. For body parts with only two levels (0 = no changes, 1 = changes, i.e., tail base, tail tip, coronary bands, and scrotum, hind limb veins), all boars were employed. In instances where body parts possess three or more levels (ears, skin, heels, and claw wall), the level exhibiting the lowest scores and the level displaying the highest scores were employed. The group with the lowest scores was decoded as 0, and the group with the highest scores was decoded as 1.

The present study investigated the effects of the boar SINS-feet scores on the SINS and body part scores of the piglets. To this end, a generalized linear mixed model was employed, in which the boar SINS group, the boar line within the boar breed as fixed effects, the sow and the boar herd as random effects, and the boar age, litter size and birth weight of the piglets as covariates were used. The investigation focused on the effects of the boar group (0 vs. 1) according to body part, utilizing a matching model in which the body part groups of the boar were used in sequence, as opposed to the SINS group of the boar.

For 20 of the 23 AI boars employed in the breeding experiment, the total breeding value and the partial breeding value for daily weight gain were available. In a generalized linear mixed model, the effect of the SINS group of the boar was calculated as a fixed effect. Concurrently, the boar line within the boar breed functioned as a fixed effect, the boar herd as a random effect, and the boar age as a covariate. The mean values were presented together with the standard error.

It is regrettable that representatives for certain breeds were only available as individual animals (Angler Saddleback) or in limited numbers (PIXDU, herdbook Large, White). Consequently, it is not possible to extrapolate the results to the entire breed.

It is imperative to note that all significance levels were Bonferroni-corrected.

## 3. Results

### 3.1. AI Boar Phenotypes

The most prevalent alterations observed in the AI boars were the absence of bristles on the ears and the tail base (65%), and ruptures in the heels (47%). The prevalence of venous congestion on the ears and detachment of the heels was observed in just over 20% of the boars (Table 1). Concurrently, substantial disparities were evident among representatives of disparate breeds, with the most pronounced values manifesting in commercial Large White and Pietrain boars. In both cases, necrosis of the tail tip was the most severe symptom in 3% of cases. A significant increase in the prevalence of bristle loss, swelling and redness was observed in boars from both breeds when compared to representatives of other breeds. When the mean values from Table 1 are set to 100%, the commercial LW reaches 179%, followed by PI (105%), DU (75%), LWH (51%), LR (42%), AS boar (31%) and PIxDU (7%). However, in the case of the AS boar, only loss of bristles on various parts of the body and cracks in the heel area were observed. In the two PIxDU boars, the only recorded findings were loss of bristles and injuries to the heels. The herdbook LW boars were found to be particularly noteworthy due to severe swelling and bleeding in the claws, as well as overall changes in the claw area. In the absence of other pertinent observations, indications of bristle loss on various parts of the body and vein congestion in the ears were noted. LR exhibited symptoms consistent with bristle loss, ear vein congestion and slight changes in the claws, similar to those observed in DU. DU also exhibited substantial reddening of the skin and excoriations. The commercial LW exhibited a markedly elevated prevalence of tail alterations in comparison to the other boars. Moreover, a substantial increase in the prevalence of these conditions was observed in the ears, skin, coronary bands and heels of the commercial Large White boars when compared to the other boars.

In any boar, the tail base was found to be devoid of exudate, necrosis and bleeding. It was established that the tail tip was devoid of both bleeding and ring constriction. The ear margins were found to be free from necrosis. No signs of exudation or necrosis were observed at the ear base, nor was there any necrosis of the ear tip. Necrosis of the skin of the boars was not detected. There was no evidence of pronounced erythema of the scrotum.

**Figure 2:**
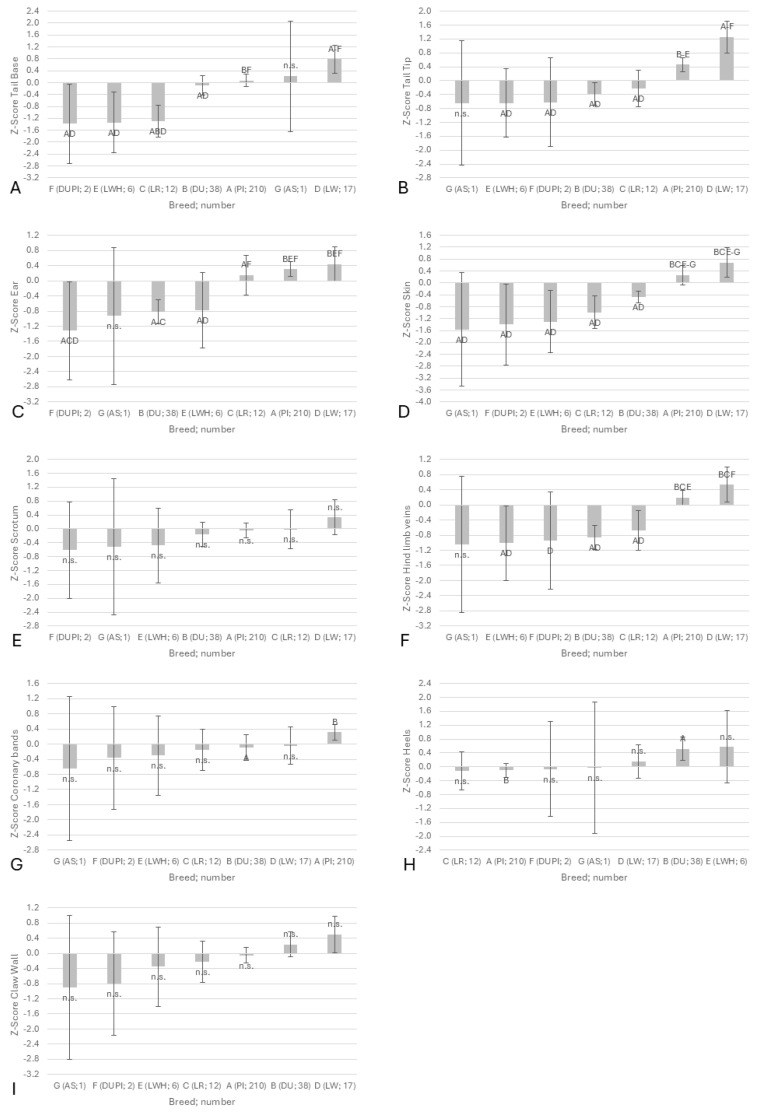
The means (columns) and the 95% confidence intervals (whiskers) of SINS-related body part scores in AI boars by breed. (**A**): tail base; (**B**): tail tip; (**C**): ear; (**D**): skin; (**E**): scrotum; (**F**): hind limb veins; (**G**): coronary bands; (**H**): heels; (**I**): claw wall. All data are subjected to Z-transformation to ensure uniform comparability. PI: Pietrain; DU: Duroc; LR: Landrace; LW: Large White, breeding company; LWH: Large White, herdbook; PIxDU: Pietrain-Duroc crossbred boars; AS: Angler Saddleback. The number of boars per breed is indicated in brackets. Letters appended to bars in the results section indicate statistical significance for boars with this particular letter; n.s. denotes non-significant difference.

The findings, summarized by body part, were Z-transformed to enhance the comparability of the results (Figure 2A–I). It is evident that due to the limited availability of data from specific breeds, a substantial 95% confidence interval was observed. With regard to the summarized body part scores, the boars from PIxDU, LWH, LR and DU were also significantly less affected than LW and PI in terms of tail base, tail tip, ears, skin and thigh veins. The coronary bands exhibited marked variations between the DU and PI boars, with DU demonstrating a statistical superiority in terms of the number of animals utilized. The severity of the claw lesions was found to be greater in the DU boars than in the PI boars. No significant differences were observed between the boars examined in terms of scrotum and claw wall. All breed effects were found to be highly significant (*p* < 0.001), with the exception of the effects on the claws (*p* = 0.054), the heels (*p* = 0.023) and the scrotum (*p* = 0.009). In addition to the impact of breed, the barn floor exhibited a substantial influence on claws and heels, particularly with regard to the separation of heels, favoring deeper litter (data not presented).

As demonstrated in Table 2, a high degree of correlation was observed between SINS signs in different body parts, with the exception of the tail base, where a low correlation was noted, and the heels, where slightly negative correlations were observed.

**Figure 3:**
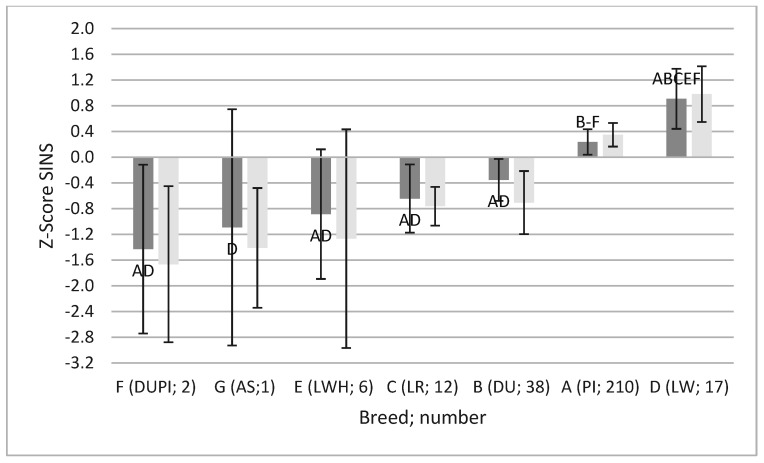
The means (columns) and the 95% confidence intervals (Whiskers) of SINS (dark bars) and SINS-feet scores (lighter bars) in AI boars of different breeds. In order to ensure the comparability of the data, it is necessary to apply the Z-transformation to all of the data. PI: Pietrain; DU: Duroc; LR: Landrace; LW: Large White, breeding company; E: Large White, herdbook; PIxDU: Pietrain-Duroc crossbred boars; AS: Angler Saddleback. The number of boars per breed is given in brackets. Letters appended to bars in the results section: statistically significant to boars with this letter. Statistical effects were observed to be consistent for both SINS and SINS-feet scores. The rank correlation between SINS and SINS-feet was found to be 0.964.

This high degree of consistency between individual body parts led to significant differences between boars of different breeds in the SINS scores, with and without consideration of heels and claw walls (Figure 3).

### 3.2. Piglet Phenotypes

The production of the piglets was exclusively from matings between sows and Duroc and Pietrain boars. The piglets presented with a high prevalence of swelling and reddening of the coronary bands and heels, hemorrhages in the claws, swollen teats and swelling of the ear veins (Table 3). The base of the tail was frequently devoid of bristles and exhibited signs of swelling. Furthermore, the tail of the subjects exhibited signs of redness in 59% of the suckling piglets, while the coronary bands demonstrated exudation in 56% of cases. Necrosis was particularly evident in the teats (12%) and coronary bands (22%). The prevalence of swelling and redness of the coronary bands was similarly high in weaned pigs as in suckling piglets (almost all animals affected). Furthermore, the teats were also observed to be reddened in 90% of weaned pigs. In 66 to 80% of weaned pigs, the tail tip was found to be free of bristles, reddened, or swollen. The alterations observed at the tail base were found to be considerably less pronounced in comparison to those seen in suckling piglets (Table 3).

**Figure 4:**
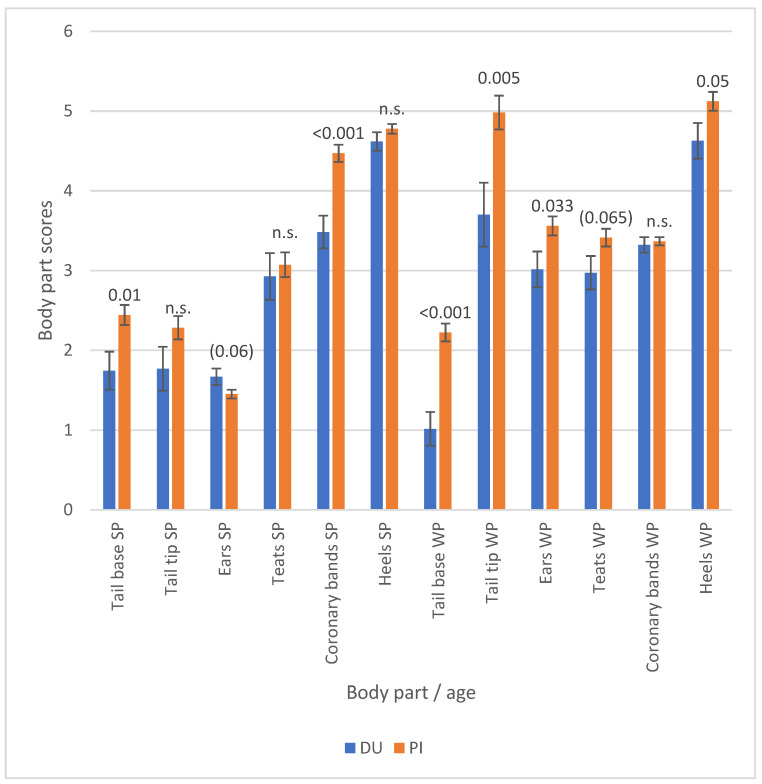
Scores for characteristics of suckling (SP) and weaned piglets (WP) by boar breed (DU = Duroc; PI = Pietrain). Numbers over the bars indicate the significance of the breed differences, with ‘n.s.’ denoting ‘not significant’.

**Figure 5:**
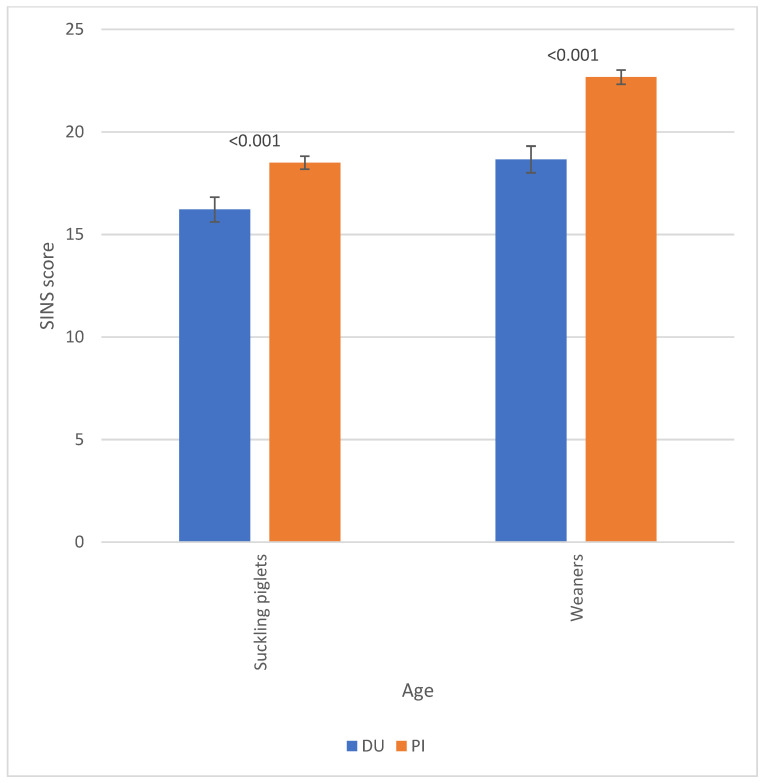
SINS scores of suckling piglets (SP) and weaners (WP) by boar breed (DU = Duroc; PI = Pietrain). Numbers over the bars indicate the significance of breed differences, with ‘n.s.’ denoting ‘not significant’.

The corresponding scores for the individual body parts, categorized by the boar breed, are displayed in Figure 4. The piglets of PI boars demonstrated higher scores for body parts in comparison to the piglets of DU boars. This phenomenon manifested most distinctly in the tail base, tail tip, ears and heels of weaners. Consequently, the corresponding total SINS scores were found to be significantly higher for suckling piglets and weaners from Pietrain boars than for the offspring of DU boars (Figure 5).

**Figure 6:**
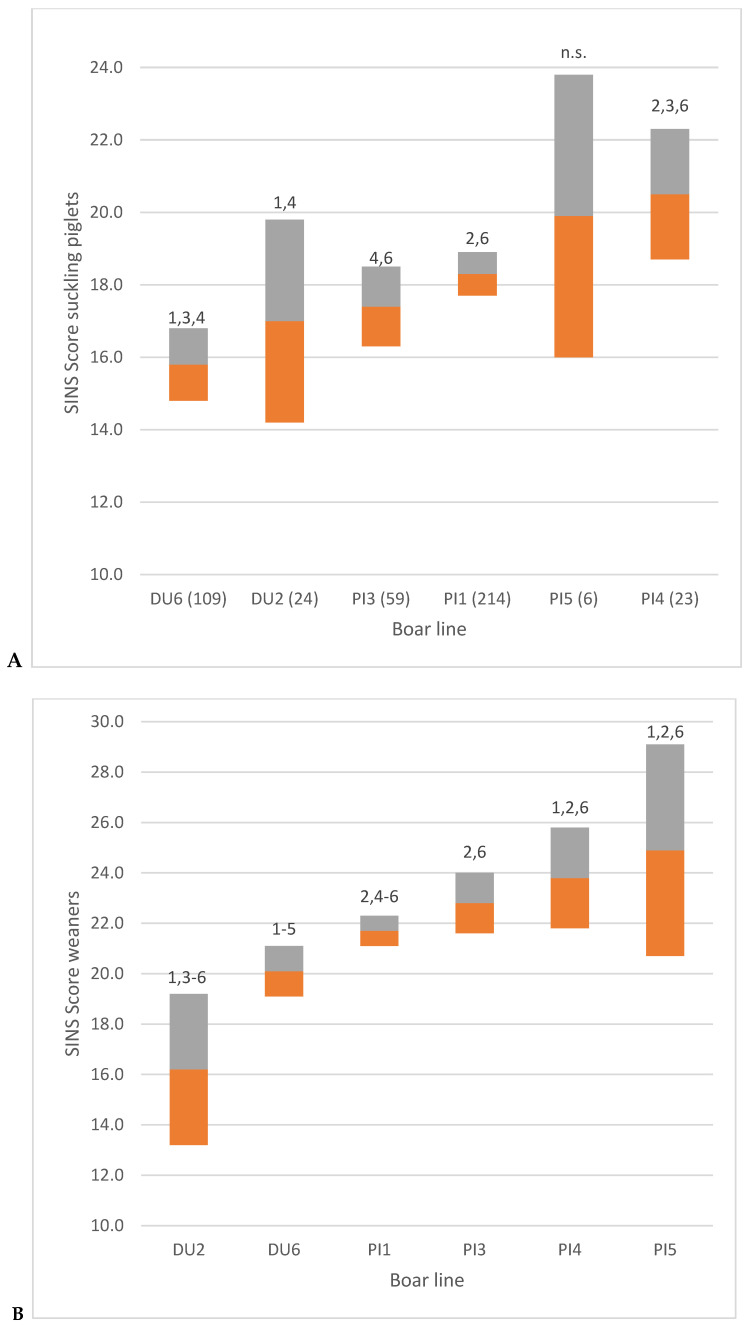
SINS score of suckling piglets (**A**) and weaners (**B**) by boar line (1–6). Numbers in brackets: number of piglets; numbers above columns: significantly different from these boar lines; DU = Duroc; PI = Pietrain; orange part of column = span between mean and lower 95% confidence interval; grey part of column = span between mean and upper 95% confidence interval.

Considerable differences were also identified between the boar lines employed within the breeds. The SINS scores of suckling piglets and weaners exhibited variation both within the Duroc and Pietrain breeds (Figure 6).

### 3.3. Associations Between SINS Scores in Duroc and Pietrain AI Boars and Their Offspring

**Figure 7:**
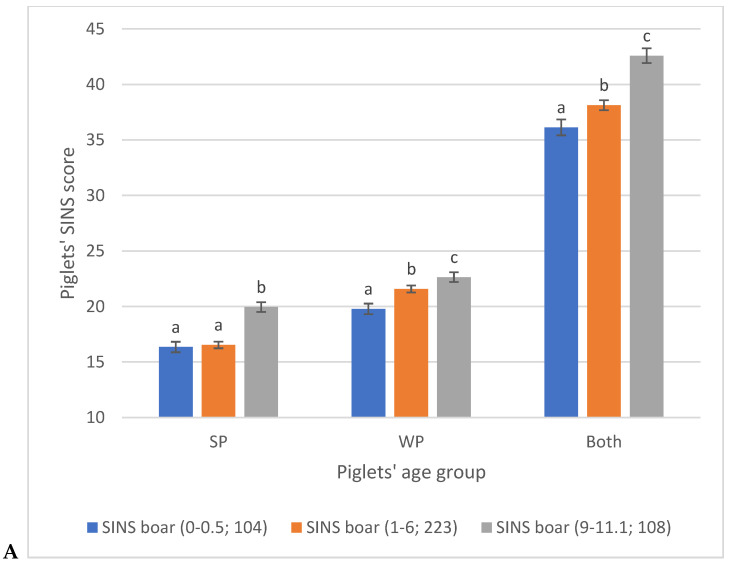

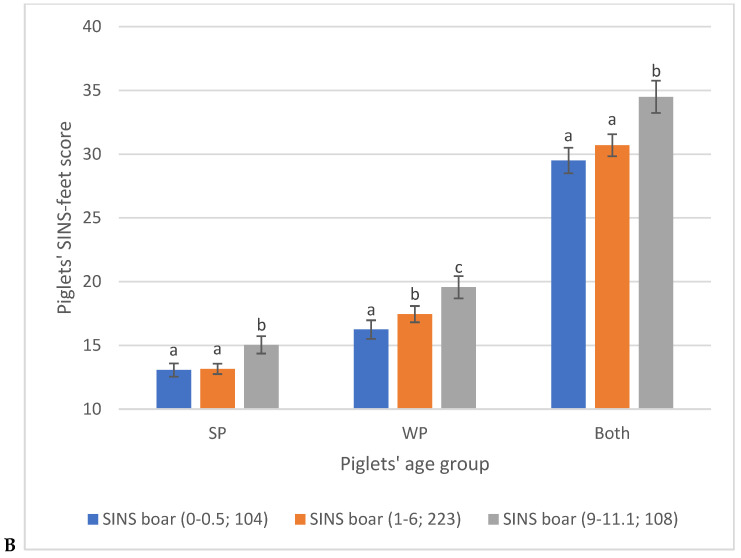
Association between the SINS score/SINS-feet score of suckling piglets and weaners, and the SINS-feet score (excluding heels and claws) of boars (DU and PI, with the boar breed considered as an effect in the model). (**A**) SINS score of the piglets including heels and claws; (**B**) SINS-feet score (i.e., without heels and claws). The figure illustrates the mean values ± standard error of the SINS scores of the offspring of boars within the 25th percentile of lowest SINS scores (blue columns), boars within the 25th percentile of highest SINS scores (grey columns), and boars with average SINS scores (orange columns). It is evident that columns marked with different superscripts exhibit statistically significant disparities. The legend delineates the range of SINS scores for boars and the number of cases available.

The boars were divided into three groups based on their SINS-feet scores (excluding claws and heels) (Figure 7). The blue columns represent the results for the boars with the lowest 25th percentile of SINS scores (SINS-feet score 0.0–0.5), while the grey line represents the results for the boars with the highest 25th percentile of SINS scores (SINS-feet score 9–11.1). The remaining boars (50%) were grouped together in the orange group (SINS-feet score 1–6). The SINS scores of suckling piglets and weaners were found to be significantly associated with the SINS scores of the boars. The offspring of boars with the 25% highest SINS scores exhibited 22% and 14% higher SINS scores than the offspring of boars with the 25% lowest SINS scores in suckling piglets and weaners, respectively. The differences between the SINS scores of the piglets were found to be largely significant (Figure 7A). With the exclusion of the claws and heels of the piglets from consideration, the SINS scores were found to be lower, yet the overall association remained comparable (Figure 7B).

**Figure 8:**
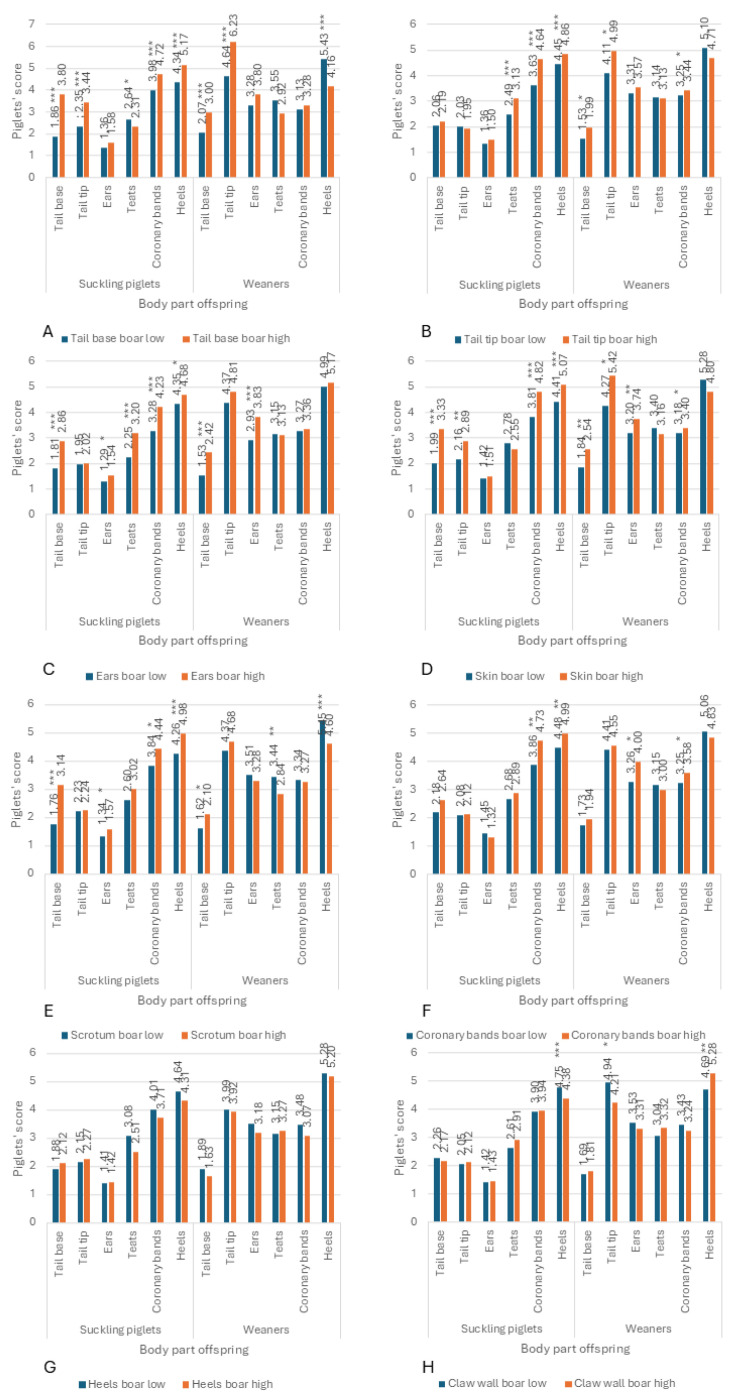
Association between SINS scores of AI boars and their offspring by body part. The bars illustrate the scores obtained for all examined body parts of suckling piglets and weaners, contingent on the group affiliation of their fathers, categorized as the 25% with the lowest (blue bars) or highest (orange bars) scores for tail base (**A**), tail tip (**B**), ears (**C**), skin (**D**), scrotum (**E**), coronary bands (**F**), heels (**G**) and claw wall (**H**). Scores and significances are given over the bars with *p* ≤ 0.05 (*), *p* ≤ 0.01 (**), and *p* ≤ 0.001 (***), respectively.

The association between the different body parts of the boars and piglets was found to vary (Figure 8). In this figure, the mean values per body part for offspring of the boars that exhibited the 25% most extreme body part scores were compared. With regard to the tail base (Figure 8A), 4 boars exhibited no alterations (tail base boar low), while 6 boars demonstrated more pronounced changes (tail base boar high). Offspring of the 6 boars with elevated tail base scores exhibited significantly elevated tail base and tail tip scores in suckling piglets and weaners. Furthermore, these offspring demonstrated higher coronary band scores in suckling piglets and higher ear scores in weaners. Concurrently, reduced teat scores were observed in suckling piglets and reduced heel scores were recorded in weaners (Figure 8A). Suckling piglets from boars with higher tail tip scores (16 boars low vs. 7 boars high) exhibited significantly higher teat, coronary band and heel scores. In weaners, the tail base, tail tip and coronary band scores demonstrated elevated levels (Figure 8B). Boars experiencing elevated levels of ear stress (5 boars low vs. 5 boars high) exhibited significantly higher tail base, ear, teat, coronary band and heel scores in suckling piglets, as well as notably higher tail base and ear scores in weaners (Figure 8C). Of the 23 boars examined, 13 exhibited no skin changes (low), while 2 demonstrated severe skin changes (high). The piglets of boars exhibiting severe skin changes demonstrated significantly elevated tail base, tail tip, coronary band and heel scores. Furthermore, weaners exhibited significantly elevated tail base, tail tip, ears and coronary band scores (Figure 8D). Of the boars, 18 exhibited no skin changes in the scrotum area, while 5 demonstrated such changes. The results of the study demonstrated that the suckling piglets of boars with changes exhibited significantly higher tail base, ears, coronary bands and heel scores, while the weaners demonstrated significantly higher tail base, but lower teats and heel scores (Figure 8E). Of the 23 boars examined, only two exhibited signs of SINS at the coronary bands. The suckling piglets exhibited significantly higher coronary band and heel scores, while the weaners demonstrated significantly higher ears and coronary band scores (Figure 8F). Of the 23 boars examined, three exhibited no alterations at the heels, while six demonstrated higher degrees of alterations. However, a comparison of the piglet scores between the two groups revealed no significant differences (Figure 8G). Furthermore, the quality of the claws of the boars was not significantly associated with the body scores of the suckling piglets, with the exception of the heels, and the weaners, with the exception of the tail tip and heels. In the case of the suckling piglets, the heels were less affected in offspring from boars with claw lesions (Figure 8H).

The tail base of the boar was found to be significantly associated with 83% of the suckling piglets’ body parts, followed by the skin and scrotum of the boar (66.7%), coronary bands (50%), hind limb veins and ears (16.7%). The condition of the heels and claws of the boar did not demonstrate any correlation with the condition of any body parts of suckling piglets. The tail tip and hind limb veins were found to be significantly associated with 50% of weaners’ body part scores, followed by the tail base, skin, coronary bands and claw wall (33.3%), and scrotum, ears and heels (16.7%).

In contrast, coronary bands, tail base and heels of the suckling piglets exhibited a significant association with the majority of the boars’ body scores (55–67%), while the ears demonstrated an association with a mere 11% of the boars’ body scores (scrotum). In weaners, the tail base and tail tip were found to be associated with body scores of the boar by 66.7% and 55%, respectively. The present study found that heels were associated with a frequency of only 11%, and that teats of weaners were not associated with any body part conditions of the boar.

**Figure 9:**
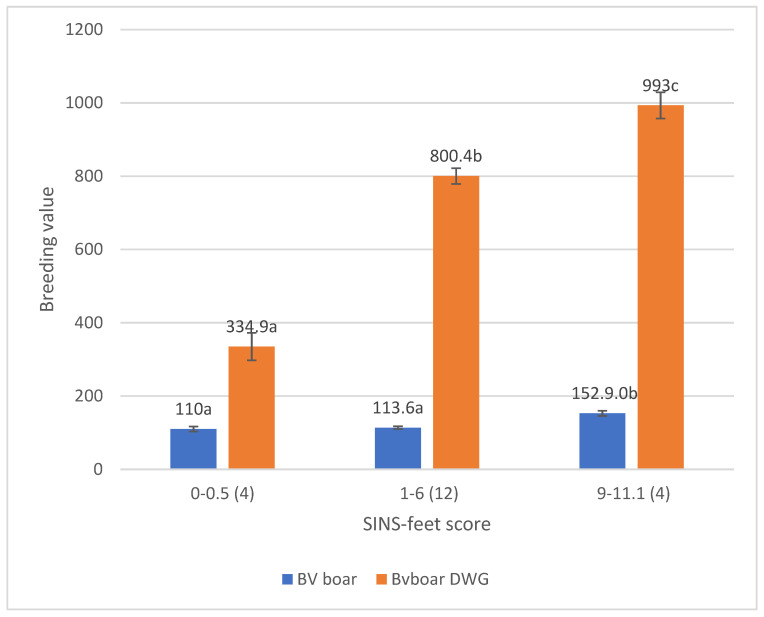
The total breeding value (BV boar) and the partial breeding value for daily weight gain (Bvboar DWG) are contingent upon the SINS status of the boar. The means and standard errors of breeding values are provided for boars with the 20% lowest SINS-feet scores (0–0.5), boars with the 20% highest SINS-feet scores (9–11.1), and the rest (SINS-feet scores 1–6). The *X*-axis denotes the range of SINS-feet scores and the number of boars in the group, which is indicated in brackets. The values expressed in the columns correspond to the breeding values. Groups with different letters are significantly different at *p* ≤ 0.05.

The total breeding value and even more the partial breeding value for daily weight gain were significantly associated with the SINS score of the boar (Figure 9).

## 4. Discussion

The results obtained demonstrated significant variations among representatives of disparate boar breeds and lines at the phenotype level of AI boars. The highest SINS scores were observed in commercial LW and Pietrain boars, which also exhibited more severe symptoms, including necrosis in approximately 3% of boars. This necrosis was otherwise largely absent in the other breeds and lines. The utilisation of Duroc boars, German Landrace boars, and herd book LW boars resulted in a conspicuously diminished SINS score. PIDU and AS were only present as individual animals, but with lowest SINS values. These results align with those of other authors [11,18]. The piglets examined exhibited a high degree of overall impact, culminating in the occurrence of necrosis in the coronary bands, teats, and tail tip at frequencies ranging from 6 to 22%. The superiority of offspring from Duroc boars over those from Pietrain boars was confirmed, along with the assumption of line differences within breeds [11,18].

Among Pietrain and Duroc boars with progeny information, the offspring of boars with medium to high SINS scores showed significantly higher SINS scores than the progeny of boars with only minor phenotypic defects. The findings were particularly well associated by the tail base, skin, coronary bands and hind limb veins of the boars, with findings in the tail base, coronary bands and heels in suckling piglets, and tail base and tail tip in weaners. Concurrently, the SINS signs of the boars exhibited a marked increase in conjunction with rising breeding values.

Evidence has been provided to demonstrate that SINS has the capacity to induce inflammatory responses and necrosis in a range of young animals, including newborn piglets [7], suckling piglets [6,11,12,18,21], and weaners [8,18,21]. The affected body parts include the tail base, tail tip, ears, teats, coronary bands, heels and claws [6,9,10,11,12]. Research has indicated that the disease is endogenous but can be modified by mechanical environmental influences at a later stage [6,7,11]. The etiology of laminitis has been demonstrated to be a response to poor floor conditions [22,23]. However, the presence of clinical lameness, claw and heel lesions can also be indicative of a generalized inflammatory process triggered by early life dysbacteriosis or stress-induced leaky gut syndrome [6,7].

The ratio between endogenous causes and mechanical environmental stress should shift towards environmental stress with increasing age [6,8]. Nevertheless, indications of SINS and associations with sow quality were also identified in fattening pigs up to this age [8,14]. Concurrently, various studies have demonstrated that the boar and boar genetics exert a substantial influence on the severity of SINS signs in their offspring [11,18]. This prompted an investigation into whether disparate AI boars exhibit varying degrees of inflammation in their external body parts, and whether these variations could be associated with the SINS score of their offspring. In the study’s preliminary phase, a total of seven distinct breeds of AI boars were utilized, with some being available only as individual animals. Consequently, the study should not be regarded as a representative sample of all breeds investigated. Nevertheless, the individual animals also provide interesting information that should be refined in future studies with larger numbers of animals. This component of the study yielded information that had not previously been available. As previously documented for suckling piglets and weaners, the absence of bristles was observed in numerous cases and on various parts of the body, sometimes in conjunction with redness and swelling, as well as congested veins. In young piglets, the absence of bristles has been demonstrated to be associated with inflammation of the hair follicles [7]. In the present study, isolated observations of hemorrhages and necrosis were recorded in boars, for example on the tail. Concurrently, substantial disparities were identified among the representatives of the diverse breeds examined. The most significant changes were observed in LW boars from commercial breeding lines, which differed considerably from the findings for herdbook LW boars and also from all other boars. The second most severely affected were representatives of the Pietrain breed. The signs exhibited in Duroc boars were also more pronounced than in German Landrace, Large White herdbook, PIxDU and Angler Saddleback boars.

This approach yielded sufficient variation in boar characteristics to facilitate the calculation of associations with the SINS data of their offspring. In the second stage of the experiment, the testing was conducted exclusively with Duroc and Pietrain boars, in order to take account of the available number of boars. A total of 23 boars were mated to 31 sows from a Topigs DL rotation cross, and 43 litters produced 435 piglets for evaluation. The distribution of inflammation and necrosis found was consistent with the prevalence described previously and also met the requirements for an association study on the piglet side. The prevalence and scores of suckling piglets and weaners were consistent with previously published data [6,8,11,12,24]. The hypothesis that Duroc boars exhibit superiority over Pietrain boars in terms of the SINS scores of their offspring, as described by Kuehling et al. [18] and Leite et al. [11], could be confirmed phenotypically when comparing the two breeds.

The significant associations observed between the inflammation and necrosis data of piglets and boars were, in principle, to be expected in this form, as it is known that there is a genetic component in boars that predisposes piglets to develop SINS [11,18]. The hypothesis is substantiated by the considerable breed disparities and the boar effects delineated, in addition to the published heritabilities. However, a significant association was identified between the body part scores of the piglets and the phenotypic scores of the tail base, skin, scrotum and coronary bands of the boars. However, no such association was identified for the heel and claw scores of the boars. This finding lends further support to the hypothesis that heels and claws, but not coronary bands, are significantly more affected by mechanical environmental influences than by endogenous SINS processes during the life of boars. Consequently, it is imperative to exclude heels and claws from the calculation of SINS scores for boars. The significant correlation between the condition of the heels of suckling piglets and 55% of the boar traits examined indicates that, at this early stage of life, the endogenous, hereditary component of the damage occurring on the heels plays a significant role alongside mechanical stress. This correlation has been confirmed in recent studies [6,11]. A significant correlation was identified between the condition of the heels of suckling piglets during the first week of life and SINS signs of piglet characteristics not directly exposed to the floor. The extant evidence would appear to suggest a correlation between significant levels of endogenous SINS stress and increased damage to the heels. The observed signs, which included swelling, redness and translucent hemorrhages, rather than open areas or cracks, suggest that endogenous stress is likely to be the primary cause of the condition of the heels, and not the other way around. In this context, a further significant finding was identified: the specificity for SINS decreased significantly with and without the body parts close to the floor during the first week of life. This finding lends further support to the hypothesis that heel problems are primarily of an endogenous nature, with mechanical factors from the environment exerting a significant influence as the individual ages [6]. The association between SINS traits in AI boars and their offspring is based on the postulated pathogenesis of SINS [9].

SINS is defined as an endogenous inflammation triggered by numerous internal and external factors and primarily affecting the intestines, liver and peripheral body parts. These factors include substances of microbial origin (microbial-associated molecular patterns; MAMPs; for MAMPs in pigs, see [25]) or endogenous origin (danger-associated molecular patterns; DAMPs [26,27]) that are recognized by macrophages and trigger the immune response. Metabolic disorders (e.g., excess lactate) and stress represent additional activating factors that emerge through the interaction of neurons and microglia in the central nervous system [28,29,30]. A significant source of MAMPs is the digestive tract. MAMPs are the result of excessive proliferation and alteration of the intestinal microbiome [31]. The underlying causes of this phenomenon have been identified as suboptimal water supply, inadequate thermoregulatory capacity, excessive protein and starch content in feed with insufficient crude fiber, and the administration of antibiotics, temporary lack of food or water, and intestinal diseases [32]. It has been established that microbial metabolites (MAMPS, LPS) are accompanied by oxidative stress and intestinal inflammation [33,34]. As demonstrated by Pearce et al. [35,36,37], disturbances in thermoregulation and suboptimal water availability can result in a reduction in intestinal blood flow and disruption of the integrity of the blood-intestinal barrier. This results in impaired expression and function of tight junctions, consequently leading to the dissolution of the intestinal surface and crypt structures. It has been demonstrated that these processes are exacerbated by a diet low in crude fiber because, in the absence of short-chain fatty acids, the intestinal microbiome metabolizes glycoproteins of the intestinal mucosa and thus the first layer of the blood-intestinal barrier [38,39,40,41]. Disruption of the blood-intestinal barrier, otherwise referred to as ‘leaky gut’, has been demonstrated to facilitate the entry of MAMPs and intestinal bacteria into the bloodstream. This, in turn, has been shown to promote inflammation in the intestine, liver and throughout the body, as well as the development of metabolic syndrome [39,42,43,44]. In sows, an increase in inflammatory markers such as ROS, IL-6 and TNFa has already been observed in mid-gestation [39]. The association of these markers with adverse outcomes, including coprostasis, fetal development stagnation, increased embryonic losses and reduced vitality of newborns, has been well documented [45,46,47,48,49,50]. Finally, the intestinal health of sows and piglets is impaired [51,52]. LPS, a significant constituent of MAMPs, has been demonstrated to be one of the most efficacious stimulants of the immune system [53]. It has been demonstrated that the entry of LPS into portal circulation through a disrupted intestinal barrier in pigs also triggers inflammation of the liver and metabolic endotoxemia [54,55,56]. Such processes have been demonstrated to result in long-term behavioral abnormalities, including tail biting [57,58]. Furthermore, it has been demonstrated that mycotoxins and their interaction with LPS promote the processes described above by enhancing the absorption of these substances from the intestine and blocking their degradation in the liver [59,60]. LPS and mycotoxins have been detected in sows’ milk and are considered to be triggers for necrosis of the tails, ears and coronary bands of suckling piglets [61,62,63,64,65].

Concurrently, substantial variations in sensitivity to SINS have been observed among offspring of different AI boars under identical environmental conditions [18]. The heritability of this SINS sensitivity has been documented at an average of around 0.3 [11] and underlying candidate genes have been identified through transcriptomic and genomic studies [16,19,20].

As demonstrated in the preceding studies on newborns, suckling piglets and weaners, there is a significant alteration in the gene expression of the liver in animals affected by SINS in comparison to those not affected by the condition [15,16]. This process has been shown to result in a shift in metabolic pathways from anabolic to inflammatory and acute phase, as evidenced by the upregulation of pivotal enzymes and mediators. The existence of associated metabolic disorders was evidenced by clinical-chemical and metabolomic studies [14,15]. The manifestation of SINS characteristics is particularly evident during the initial week of life, with a subsequent peak observed around the fourth week [21]. The fluctuations in SINS signs during the initial week of life are directly proportional to the initial surge in metabolic turnover, which occurs in the first days of life. This decline continues until the tenth day of life, as demonstrated in the study by Studzinski [66]. The study demonstrates that metabolic requirements per kilogram of body weight are particularly elevated during the initial week of life. A further challenge is posed by the weight range between 10 and 35 kg, wherein the highest metabolic activity, particularly in the liver and intestines, is concomitant with an immune system that has not yet reached full development [67,68,69]. Although the highest growth performance is achieved in the 75 to 90 kg body weight range, currently with average daily gains of over 1200 g/animal in certain lines [70], this is significantly less per kg body weight than in younger piglets.

From approximately 12 months of age, a stagnation in daily weight gain becomes evident [67], and the metabolism’s primary function shifts to the maintenance of body weight and fat deposition. The significant metabolic activity of piglets, which is particularly evident during growth, is not considered relevant in the present study of AI boars used for insemination that are at least 13.5 months old (with an average age of 26 months). Moreover, the production of semen exerts no considerable influence on metabolism in mammals, including boars [71]. Nevertheless, substandard semen quality remains the primary cause of culling Pietrain boars at European insemination stations [72]. However, at this age, AI boars still demonstrate significant meatiness, particularly those of the Pietrain breed. As demonstrated by Hermesch [73], a negative correlation has been demonstrated between high meatiness and meat quality, fattening performance, feed efficiency, and reproduction. Health-related indicators, such as lameness and claw diseases, as well as behavioral and welfare-related indicators, such as high breeding culling rates and shorter life spans, are also affected [74,75,76,77]. The present studies demonstrated that pigs from performance-selected lines, characterized by increased fattening performance and meatiness, respond to both pathogens and environmental stress by increasing the expression of genes associated with inflammation (e.g., IL-1β, TNF-α and toll-like receptors) [78].

In addition, these lines exhibited elevated levels of oxidative stress markers and diminished antioxidant enzyme activity in comparison to less selected lines. Elevated acute phase proteins (e.g., Pig-MAP, CRP, haptoglobin) and oxidative stress markers (ROS, TBARS, protein carbonyls) were indicative of transport, heat stress or feed change [79]. Furthermore, certain mediators and genes were found to be overexpressed in piglets with SINS in comparison to those without SINS [15,16].

The presence of meatiness has been demonstrated to disrupt homeostasis. This phenomenon can be attributed to the heightened metabolic activity associated with increased muscle mass. This heightened activity results in an augmented oxygen demand, an elevated blood supply to muscle tissue, and an elevated energy turnover. Poor capillarization and large fiber diameters have been demonstrated to result in oxygen deficiency, particularly during periods of stress and exertion. It has been demonstrated that this process gives rise to elevated levels of lactate, a by-product of anaerobic glycolysis. It has been demonstrated that lactate has the capacity to induce acidosis through a reduction in the pH of the blood and muscles. This results in the release of calcium from muscle fibers, leading to uncontrollable contractions. Concurrently, the hypothalamic–pituitary–testicular axis (HPTA) and the toll-like receptor (TLR) pathway are activated, thereby triggering an inflammatory response [80]. Meat breeds (e.g., Pietrain) are particularly affected by these conditions due to their high proportion of type IIb fibers [81,82,83,84]. Elevated lactate levels have been demonstrated to activate HIF-1α even under normoxic conditions, resulting in substantial metabolic aberrations [85]. Observations have been made of the binding of lactate to GPR81/HCAR1 on fat cells, thereby inhibiting lipolysis and influencing the energy distribution between glucose and fat metabolism [86]. Serum lactate levels of 5.1–8.0 mmol/L; represent severe hyperlactatemia, leading to intracellular acidosis, reduced muscle contraction, energy crisis, heat stress response, decreased performance and reduced well-being [84]. Levels of lactate exceeding 8.0 mmol/L have been demonstrated to induce severe metabolic derailment, also known as acidosis. As Gladden [87] hypothesizes, calcium and mitochondrial dysfunction disrupt cellular homeostasis, thereby activating inflammatory signaling pathways. Lactate has been demonstrated to induce the expression of vascular endothelial growth factor (VEGF) in macrophages [88,89,90,91]. VEGFA is known to be regulated by proinflammatory cytokines, including TNF-α, IL-1β and IL-6. It has been established that another typical feature of inflammation and ischemia is that the expression of VEGFA is triggered by the hypoxia-induced factor (HIF-1α), which is activated in tissue damage. VEGF has been demonstrated to promote vascular permeability, thus facilitating the entry of neutrophils and macrophages into the tissue and causing edema, a feature of acute inflammation [92,93]. VEGFA exerts a chemotactic effect on monocytes and macrophages, thereby stimulating the immune response in inflammatory foci. VEGFA has been observed to be overexpressed in chronic inflammation, such as in cases of chronic intestinal diseases and rheumatoid arthritis [94]. It has been identified as a key factor in the inflammatory response in SINS [16]. The authors discuss VEGFA as a potential mediator of hypoxia-induced angiogenesis, immune cell recruitment and inflammatory microvascular changes. In contrast, Pietrain-boars have been observed to exhibit higher levels of lactate at rest in comparison to their crossbred counterparts [95]. In situations characterized by stress or during the process of semen collection, elevated levels of lactate, exceeding 10 mmol/L, have been observed, resulting in the onset of acidosis, metabolic imbalances, and compromised animal welfare [96].

It is regrettable that the study did not include further clinical chemistry values, primarily due to considerations of animal welfare. Nevertheless, the inflammation observed in this study, particularly in commercial Large White and Pietrain boars, may be attributable to meatiness and lactate levels. This issue must be the subject of further investigation. As demonstrated in the relevant literature, elevated daily weight gains have also been shown to be conducive to the production of lactate. This phenomenon can be attributed to the fact that such gains engender elevated levels of performance and energy requirements, in addition to those of oxygen. The existence of leptin resistance in animals with a high consumption of lean meat has been demonstrated to promote metabolic imbalances and inflammation [97,98,99]. The combination of signs of SINS in AI boars with their respective clinical chemical values would be a fascinating avenue for future research. In the present study, boars from breeds and lines with high performance were found to be significantly more affected by signs of SINS. This was also evident in a positive correlation between SINS score and breeding value within the Pietrain breed. In contrast, Leite et al. [11] found no correlation between the meat-fat ratio and SINS scores in piglets. The reasons for this discrepancy remain to be elucidated. The breeding values of AI boars exhibited a stronger correlation with their SINS phenotypes than with the SINS phenotypes of piglets. Concurrently, the associations between SINS phenotypes of AI boars and piglets were found to be significant. Although higher-performing AI boars exhibited an increased propensity for inflammation, which can evidently be transmitted to piglets [11,18], this inflammatory effect in piglets could be partially compensated for by the sow. This would serve to reduce the association between boar performance and the SINS scores of their offspring.

The evaluation of SINS in AI boars can be accomplished with relative ease and minimal effort. The method is non-invasive and animal-friendly, yet still allows the identification of AI boars that are particularly susceptible to SINS, including with regard to the transmission of this susceptibility to offspring. The integration of SINS screening into the pre-selection of AI boars appears to be a favorable proposition, provided that the body parts exposed to particular mechanical stress, i.e., heels and claws, are excluded from the analysis. Moreover, no substantial correlation with age could be ascertained. The SINS score is thus composed of multiple observations that can be distinctly categorized as present or absent (e.g., redness, swelling, bleeding, etc.) and documented as binary data.

## 5. Conclusions

The present results indicate that, in the context of Swine Inflammation and Necrosis Syndrome (SINS), endogenous processes are not only heritable and affect the sensitivity of offspring, but can also manifest in the phenotype of AI boars. This finding lends weight to the hypothesis of an inherent predisposition in AI boars. Selection decisions among otherwise comparable boars can therefore be guided by specific phenotypic indicators, particularly the condition of the tail base and coronary bands. Using these criteria when selecting AI boars may reduce their offspring’s susceptibility to inflammation and necrosis, thereby promoting animal health and welfare.

## Figures and Tables

**Table 1:** Prevalence of signs of inflammation and necrosis in AI boars by breed (%).

		Large White Commercial	Pietrain	Duroc	Landrace	Large White Herdbook	PIDU	Angler Saddle-Back	Overall
Body Part	Characteristic	a ^1^ (17) ^2^	b (210)	c (38)	d (12)	e (6)	f (2)	g (1)	
Tail base	no bristles	94.7 (b–f) ^3^	75.2 (a,d–f)	68.0 (a,d–f)	6.6 (a–c,g)	9.1 (a–c,g)	3.2 (a–d,g)	94.6 (d–f)	47.4 ± 0.269
	Swelling	7.3	4.1	4.2	2.6	2.4	3.1	1.8	3.3 ± 0.036
	Redness	10.8	1.1	1.6	0	0	0	0	0.0 ± 0.012
Tail tip	no bristles	89.2 (b–g)	27.1 (e)	17.6 (e)	21.5	2.4	2.9	2.2	14.0 ± 0.130
	Swelling	7.1	4.2	2.6	2.4	2.1	3	1.7	3.0 ± 0.033
	Redness	10.6	6.8	0	0	0	0	0	0.0 ± 0.003
	Exsudate	6.8	3.1	3	2.8	2.8	3	2.5	3.7 ± 0.036
	Necrosis	3.2	3	2.9	2.7	2.6	3	2.1	2.8 ± 0.031
Ears	no bristles (low)	21.5 (b,g)	38.6 (c,g)	62.9 (g)	49.3 (g)	30 (g)	50.9	99.8 (c–e)	64.8 ± 0.872
	no bristles (high)	78.3 (c,e–g)	60.5 (c,f,g)	31 (a,b,f,g)	46.1 (f,g)	29.3 (a,f,g)	0 (a–e,g)	100 (a–f)	51.2 ± 8.53
	ear margin no bristles (low)	59.3 (b,e–g)	31.7 (a,e–g)	23.4 (f,g)	26.7 (f,g)	9.2 (a,b,f)	0 (a–d,g)	100 (a–f)	33.3 ± 4.63
	ear margin no bristles (high)	16.1	10.7 (c–g)	2.3	0	0	0	0	0.0 ± 0.022
	ear base redness	26.1	18.9 (e)	10	21.5	3.5	3.2	2.4	8.7 ± 0.087
	venous congestion (low)	59	67.3 (c,e–g)	16.8 (a,b,d)	63.8 (c,f,g)	30.9 (bc,f,g)	2.7 (a,b,d)	3.1 (a,b,d)	24.8 ± 0.198
	venous congestion (high)	23.7	16.2	2.3 (a,b)	17.4	7.3	3	1.4	6.7 ± 0.068
Skin	no bristles	72.1 (c–g)	67.7 (c–g)	26.9 (a,b,d–g)	5.6 (a,b)	0 (a–c)	0 (a–c)	0 (a–c)	0.5 ± 0.267
	Redness	2.9	2.9	4.1	2.9	2.8	2.9	2.9	3.0 ± 0.034
	rhaghades	3.8	6.9	15.5	6.3	2.2	3.4	1.2	4.2 ± 0.046
Scrotum	Swelling	35.3	21.3	6.3	17	7.8	0	0.2	3.4 ± 0.250
Coronary bands	Swelling	3.3	5.6	10.1	3	3.7	3.1	2.6	4.0 ± 0.044
	Redness	7.3	12.2 (f,g)	4.3	5.4	6.8	0 (b)	0 (b)	0.5 ± 0.268
	exudation	12.8	3.2	3.2	2.7	2.7	3.1	2	3.5 ± 0.038
Heels	Swelling	11.7	11.5 (d)	6.3	2.3 (b)	37	2.9	1.5	6.4 ± 0.066
	Bleeding	10.1	6.7	2.1	2.6	13.4	2.7	2.6	4.5 ± 0.048
	Rupture	66.3 (f,g)	43.2 (f,g)	49.3 (f,g)	41.1 (f,g)	35.2 (f,g)	0.1 (a–g,g)	99.5 (a–f)	40.7 ± 1.775
	detachment	74.7 (b–e,g)	21.7 (a,g)	35.1 (a,e,g)	10.2 (a,c)	18.4	50.7	0.6 (a,b)	20.4 ± 0.149
Claw wall	Cleft	28.2	9.1	18.5	8.4	18.8	0	0	1.6 ± 0.329
	Bleeding	2.8	3.2	3	3	3.1	2.8	3.3	3.0 ± 0.034
Hind limb	vein congestion	78 (c–g)	61.8 (c–g)	7.8 (a,b)	21.4 (a,b)	2.9 (a,b)	2.9 (a,b)	2.8 (a,b)	14.2 ± 0.134

^1^ Letter representing the breed; ^2^ number of boars; ^3^ this breed is significantly different from the breeds with the respective letters (*p* ≤ 0.05).

**Table 2:** Spearman’s Rho correlations between different body parts of AI boars.

	TT	Ear	Skin	Scrotum	Veins	CB	Heel	Claw
TB	0.393	0.536	0.464	0.536	0.393	0.393	0.036	0.429
TT		0.821 *	0.929 **	0.857 *	1.000 **	0.893 **	−0.321	0.857 *
Ear			0.857 *	0.929 **	0.821 *	0.821 *	−0.107	0.750
Skin				0.857 *	0.929 **	0.964 **	0.000	0.964 **
Scrotum					0.857 *	0.786 *	−0.179	0.821 *
Veins						0.893 **	−0.321	0.857 *
Coronary							−0.107	0.893 **
Heel								0.143

TB: tail base; TT; tail tip; Veins: Hind limb vein congestion; CB: coronary bands; *: *p* < 0.05; **: *p* < 0.01.

**Table 3:** Prevalence of SINS signs at different body parts of suckling piglets and weaners in percentage.

Body Part	Characteristic	Suckling Piglets	Weaners
Tail base	Loss of bristles	71.0	58.0
	Swelling	62.2	32.0
	Redness	15.3	37.0
	Exudation	0.7	0.0
	Necrosis	0.0	0.0
	Bleeding	0.0	0.0
Tail tip	Loss of bristles	27.8	79.3
	Swelling	29.2	65.6
	Redness	59.9	72.2
	Exudation	2.1	30.4
	Necrosis	0.7	5.9
	Ring constriction	0.9	0.0
	Bleeding	0.2	0.0
Ears	No bristles, low	19.1	48.0
	No bristles, high	0.0	34.4
	Margin, no bristles, low	7.2	24.7
	Margin, no bristles, high	0.0	2.8
	Ear base redness	14.9	15.5
	Ear base exudation	0.0	0.0
	Ear base necrosis	0.0	0.7
	Ear margin necrosis	0.0	24.0
	Ear tip necrosis	0.0	59.8
	Vein congestion low	89.6	86.8
	Vein congestion high	11.3	26.8
	Injuries	60.8	90.8
Teats	Swelling	86.0	61.1
	Redness	31.9	90.1
	Scabs	9.3	3.8
	Necrosis	12.0	3.3
Coronary bands	Swelling	99.8	100.0
	Redness	97.3	98.6
	Exudation	55.9	12.7
	Necrosis	22.1	1.6
Heels	Swelling	98.9	99.8
	Bleeding low	57.2	80.5
	Bleeding mean	86.3	0.0
	Blutung high	60.6	0.0
	Ablasions	5.4	89.6
Claw wall	Bleeding	59.7	100.0
	Ablasion	0.7	1.4
	Split	1.4	0.0
	Cleft	0.7	34.1

## Data Availability

The datasets used and analysed during the current study are available from the corresponding author on reasonable request; the data are not publicly available due to privacy restrictions.

## Abbreviations

The following abbreviations are used in this manuscript:

SINS	Swine inflammation and necrosis syndrome
PI	Pietrain
DU	Duroc
LW	Large White, commercial lines
LWH	Large White, herdbook
LR	Landrace
AS	Angler Saddleback
SP	Suckling piglets
WP	Weaned piglets
